# Complementary traditional Chinese medicine use in Children with cerebral palsy: a nationwide retrospective cohort study in Taiwan

**DOI:** 10.1186/s12906-017-1668-5

**Published:** 2017-03-14

**Authors:** Hou-Hsun Liao, Hung-Rong Yen, Chih-Hsin Muo, Yu-Chen Lee, Mei-Yao Wu, Li-Wei Chou, Mao-Feng Sun, Tung-Ti Chang

**Affiliations:** 1 Graduate Institute of Chinese Medicine, School of Chinese Medicine, College of Chinese Medicine, China Medical University, Taichung, Taiwan; 2 Department of Chinese Medicine, Dalin Tzu Chi Hospital, Buddhist Tzu Chi Medical Foundation, NO. 2, Min-Sheng Road. Dalin Town, Chiayi, Taiwan, Republic of China; 3 Department of Chinese Medicine, China Medical University Hospital, 2 Yude Road, North District, Taichung, 404 Taiwan; 4 Research Center for Chinese Medicine & Acupuncture, China Medical University, Taichung, Taiwan; 5 Research Center for Chinese Herbal Medicine, China Medical University, Taichung, Taiwan; 6 Research Center for Traditional Chinese Medicine, Department of Medical Research, China Medical University Hospital, Taichung, Taiwan; 7 Management Office for Health Data, China Medical University Hospital, 2 Yude Road, North District, Taichung, 404 Taiwan; 8 Graduate Institute of Acupuncture Science, College of Chinese Medicine, China Medical University, 91 Hsueh-Shih Road, North District, Taichung, Taiwan; 9 Department of Physical Medicine and Rehabilitation, China Medical University Hospital, 2 Yude Road, North District, Taichung, Taiwan; 10 School of Post-Baccalaureate Chinese Medicine, College of Chinese Medicine, China Medical University, Taichung, Taiwan

**Keywords:** Acupuncture, Cerebral palsy, Rehabilitation, Epidemiology, Medical expenditure, National health insurance research database, Traditional Chinese medicine

## Abstract

**Background:**

Complementary traditional Chinese medicine (TCM) has been used to treat patients with cerebral palsy (CP). However, large-scale surveys examining its use in the treatment of CP and associated disorders are lacking.

**Methods:**

We enrolled 11,218 patients ≤ 18 years of age with CP in the Taiwanese National Health Insurance Research Database from 1995 to 2011. Patients were categorized as TCM users (*n* = 6,997; 62.37%) and non-TCM users (*n* = 4,221; 37.63%) based on the inclusion of TCM in their treatment plan.

**Results:**

Children with higher proportions of complementary TCM use were male, younger, and lived in urbanized areas. Most TCM users (*n* = 5332, 76.2%) visited TCM outpatient departments more than 20 times per year. In both groups, the three most common reasons for clinical visits were problems of the nervous system, respiratory system, and digestive system. Acupuncture was commonly used in problems of injury, musculoskeletal system and connective tissue, and nervous system. Chinese herbal medicine was used to improve the primary symptoms of CP in patients, as well as its associated disorders. The incidence rate ratios in allergic rhinitis, dyspepsia, menstrual disorders, and musculoskeletal system and connective tissue diseases among TCM users were significantly higher than non-TCM users. Although patients receiving complementary TCM therapies had higher medical expenditure for utilizing outpatient clinical consultations, their medical costs for visiting ER and hospitalization were significantly lower than that of non-TCM user within one year of the diagnosis of CP.

**Conclusion:**

This study was a large-scale survey to characterize patterns of complementary TCM use among children with CP. The complementary use of TCM in children with CP was considerably high. Future clinical trials and basic researches can be developed based on the findings of this study.

## Background

Infantile cerebral palsy (CP) refers to a non-progressive syndrome characterized by hypoxia of the underdeveloped brain of infants or children below the age of two (prenatal or perinatal period), thereby inducing postural and motor disabilities [1]. There are many causes of CP, and most cases are believed to be due to prenatal factors. The most common risk factors for CP are prematurity, followed by intrauterine growth restriction, intrauterine infection, antepartum hemorrhage, severe placental pathology, and multiple pregnancy [2]. It is the most common motor disability in childhood with a prevalence of approximately 2 to 4 cases per 1000 children [3].

Current treatment including physical therapy and occupational therapy plays an important role in treating children with CP [4]. In addition to abnormalities of motor activity and posture, children with CP often have other disorders of cerebral function, including intellectual disability or specific learning disabilities, behavioral and emotional disorders, seizures and impaired vision and speech [5]. Therefore, teams consisting of the family and medical staff are necessary to maximize children's social and emotional development, communication, education, nutrition, mobility, and independence in daily activities [6, 7].

The integration of traditional Chinese medicine (TCM) has been widely practiced in Taiwan. It has been used to treat various pediatric diseases such as asthma [8], atopic dermatitis [9], rhinosinusitis [10, 11], diabetes [12], precocity [13], and cancer [14]. A few clinical [15] or animal [16] studies have reported treating CP with TCM, especially by acupuncture. However, there is a lack of large-scale, population-based, epidemiological analyses regarding the utilization patterns of complementary TCM for children with CP.

Since 1995, the majority of the total population in Taiwan (23 million people) has been enrolled in the mandatory National Health Insurance (NHI) program [17]. The practice of TCM has been reimbursed by the NHI program since 1996. Although the broad definition of TCM includes Chinese herbal medicine, proprietary Chinese medicine, acupuncture, moxibustion, manipulation, and Qi management, only the following three major modalities have been covered: (1) Chinese herbal medicine manufactured by GMP-certified pharmaceutical companies (concentrated scientific TCM granules), (2) acupuncture/moxibustion (including acupuncture, moxibustion and cupping therapy) and (3) Chinese orthopedic traumatology therapy (including manipulative therapy, acupressure, and tuina massage) [18]. All claims data were collected in the National Health Insurance Research Database (NHIRD).

We investigated the characteristics of adjunctive TCM use in children with CP by analyzing Taiwan’s NHIRD. This dataset comprehensively included all children who were clinically and radiographically confirmed to have CP with long-term follow-up, thus reducing the potential for sampling bias. This study was important in setting the foundation for understanding the patterns of complementary TCM utilization. The results of this study provided useful information for those involved in the healthcare of children with CP.

## Methods

### Data source

Taiwan launched the NHI program in March 1995, and by 2015, it covered more than 99% of Taiwanese residents [17]. Since 1995, the NHI program has reimbursed nearly all of the necessary Western medical services and included TCM services in 1996. The choice to utilize TCM or Western medicine belongs to the patient and is not influenced by the insurer. Only licensed TCM doctors are qualified for reimbursement. The large computerized NHI database (NHIRD; http://nhird.nhri.org.tw/) was provided by the National Health Insurance Administration and maintained by the National Health Research Institutes of Taiwan. The registry comprises de-identified information regarding medical care facilities, specialties, gender, birth dates, visit dates, prescriptions, health management, costs and diagnostic codes based on the International Classification of Disease, 9th Revision, Clinical Modification (ICD-9-CM). The NHIRD also established a “registry for catastrophic illnesses patient database (RCIPD)", which included approximately 30 disease categories such as cancer, schizophrenia, end-stage renal disease, multiple sclerosis and CP. Catastrophic illness certificates were administered to children with CP who had completed a clinical and neuroimaging evaluation, followed by a thorough and routine review by pediatricians or rehabilitation physicians appointed by the NHI Administration. Thus, the diagnosis of CP in children participating in this study was highly reliable.

### Study subjects and variables

**Fig. 1:**
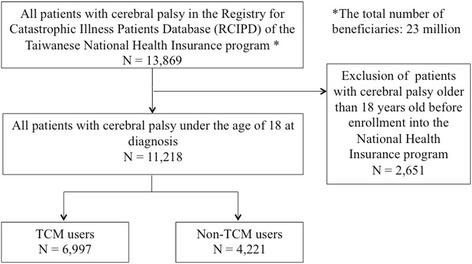
Flow recruitment chart of children with cerebral palsy from the registry for catastrophic illnesses patient database (RCIPD) of the Taiwanese National Health Insurance Research program from 1995 to 2011

The flow chart for the selection of CP cases is illustrated in Fig. 1. Of all 23 million enrollees of the NHI program, patients under the age of 18 (*n* = 11,218) at the time of diagnosis of CP (ICD-9-CM code: 343) in the RCIPD of NHIRD were included in this study. They were then followed-up until the end of 2011. After an accurate diagnosis of CP, the children who consulted with TCM doctors were grouped as TCM users (*n* = 6,997), while the others were grouped as non-TCM users (*n* = 4,221). The demographic characteristics and claims data of this study cohort were collected and analyzed.

The urbanized residential areas of all individuals were divided into two groups, with urban areas more highly represented than rural areas. The urbanization of residential areas has been described previously [19, 20]. In brief, the residential areas of Taiwan were divided into 4 levels of urbanization based on population density (people/km^2^), the ratio of the population with varying educational levels, the ratio of elderly people, the ratio of agricultural workers, and the number of physicians per 100,000 people. Levels 1 and 2 of this urbanization were defined as urban areas, while levels 3 and 4 were classified as rural areas.

The concentrated scientific TCM granules included TCM herbal formulas and single herbs. Therapeutic actions and indications of TCM prescriptions were recorded based on the TCM theory [21]. The core prescription patterns were analyzed as described previously [22, 23]. In brief, an open-sourced freeware NodeXL (http://nodexl.codeplex.com/) was applied to investigate the core patterns of Chinese herb medicine for the treatment of patients with CP, and the most common two herbal combinations were applied in this network analysis. The thicker line width, defined as counts of connections between formulas and herbs, indicated the more significant prescription patterns in the network.

The medical expenditure of utilizing emergency room (ER) service, outpatient clinical care and hospitalization between patients with and without TCM treatment within one year after CP was diagnosed were calculated in New Taiwan dollars.

### Ethics statement

The NHIRD was provided by the National Health Insurance Administration and managed by the National Health Research Institutes, Taiwan. All of the datasets were de-identified and encrypted to protect enrollees’ privacy. Therefore, it was not possible to identify individual patients in any way. This study was approved by the Research Ethics Committee of China Medical University and Hospital (CMUH104-REC2-115).

### Statistics

The data were analyzed by using SAS software, version 9.2 (SAS Institute Inc., Cary, NC, U.S.A.). We used the chi-square test to compare categorical variables and the *t*-test to compare continuous variables. We estimated the incidence rate ratio and 95% confidence intervals (CIs) by using the Poisson regression. A P value <0.05 was defined as statistically significant.

## Results

We identified 11,218 patients under the age of 18 who were diagnosed with infantile CP with catastrophic illness certificates (Fig. 1). Among these children, 62.37% (*n* = 6,997) were TCM users, while 37.63% (*n* = 4,221) were not TCM users. Some proportional differences were found between TCM and non-TCM users in age, sex, urbanization, and annual outpatient clinical visits (Table 1). Children with higher proportions of TCM use were male, younger (age 0–2 and 3–5), and resided in urbanized areas. Furthermore, most TCM users (*n* = 5,332, 76.2%) visited TCM outpatient departments more than 20 times per year. By contrast, the rate of annual outpatient visits was lower in non-TCM users than in TCM users. However, it has to be mentioned that these small differences may not be clinically significant but may be statistically significant given the large sample size.

**Table 1:** Demographic characteristics between TCM and non-TCM users among children with cerebral palsy from 1995–2011

	TCM user		Non-TCM user		
	*N* = 6997		*N* = 4221		
	N	%	N	%	*p*-value
Sex^a^					0.008
Girl	2851	40.8	1827	43.3	
Boy	4146	59.3	2394	56.7	
Age^a^, year					<0.0001
0–2	3086	44.1	1444	34.2	
3–5	1845	26.4	896	21.2	
6–12	1567	22.4	1281	30.4	
13–18	499	7.13	600	14.2	
Urbanization^a^					<0.0001
Urban	4132	59.0	2322	55.0	
Rural	2865	41.0	1899	45.0	
Annual outpatient clinical visit^a^					<0.0001
< 5	113	1.61	318	7.53	
5–9	386	5.52	517	12.3	
10–19	1166	16.7	1103	26.1	
20+	5332	76.2	2283	54.1	
Mean (SD)^b^	41.5	(28.3)	26.7	(21.2)	<0.0001

^a^Chi-square test; ^b^Student’s *t*-test

We further analyzed the frequency distribution of TCM and non-TCM visits by major disease category/diagnosis in children with CP (Table 2). In both groups, the three most common causes of clinical visits were problems of the nervous system (46.6% in TCM users versus 45.6% in non-TCM users), respiratory system (26.4% in TCM users versus 27.6% in non-TCM users), and digestive system (6.12% in TCM users versus 6.20% in non-TCM users).

**Table 2:** Frequency distribution of TCM and non-TCM visits by disease categories/diagnosis among children with cerebral palsy

	TCM user		Non-TCM user	
	Number of clinical visits =3214649		Number of clinical visits =1104382	
Disease (ICD-9-CM)	Visits no.	%	Visits no	%
Infectious and parasitic disease (001–139)	87845	2.73	50181	4.54
Neoplasms (140–239)	3992	0.12	1726	0.16
Endocrine, nutritional and metabolic disease and immunity disorder (240–279)	16310	0.51	5527	0.50
Blood and blood-forming organs (280–289)	2894	0.09	1208	0.11
Mental disorder (290–319)	195321	6.08	53797	4.87
Nervous system (320–389)	1496602	46.6	503782	45.6
Circulatory system (390–459)	21600	0.67	5241	0.47
Respiratory system (460–519)	847770	26.4	304833	27.6
Digestive system (520–579)	196656	6.12	68418	6.20
Genitourinary system (580–629)	16555	0.51	5870	0.53
Complications of pregnancy, childbirth and the puerperium (630–676)	430	0.01	74	0.01
Skin and subcutaneous tissue (680–709)	56605	1.76	24637	2.23
Musculoskeletal system and connective tissue (710–739)	30253	0.94	7420	0.67
Congenital anomalies (740–759)	56202	1.75	20061	1.82
Certain conditions originating in the perinatal period (760–779)	26011	0.81	7171	0.65
Symptoms, signs and ill-defined conditions (780–799)	102187	3.18	28918	2.62
Injury and poisoning (800–999)	57416	1.79	15518	1.41

We also analyzed the complementary TCM treatment options that the patients received (Table 3). Chinese herbal medicine was mainly used for treating symptoms related to nervous system, respiratory system, signs and ill-defined conditions. Acupuncture was mainly used for treating problems of injury, musculoskeletal system and connective tissue disorder, and nervous system. Chinese orthopedic traumatology was mainly used for symptoms related to nervous system, mental disorder, and injury.

**Table 3:** Frequency distribution various TCM therapies by major disease categories/diagnosis among children with cerebral palsy

Disease (ICD-9-CM)	CHM	COT	ACU	CHM + COT	CHM + ACU	CHM + COT + ACU
Infectious and parasitic disease (001–139)	0.38	0.31	0.10	0.73	0.13	0.35
Neoplasms (140–239)	0.04	0.06	0.05	0.01	0.07	0.04
Endocrine, nutritional and metabolic disease and immunity disorder (240–279)	0.29	0.13	0.00	0.02	0.00	0.21
Blood and blood-forming organs (280–289)	0.13	0.02.	0.00	0.05	0.00	0.08
Mental disorder (290–319)	4.07	7.73	0.21	5.07	0.26	5.21
Nervous system (320–389)	31.3	76.6	22.5	67.6	27.2	47.7
Circulatory system (390–459)	0.96	2.32	1.47	3.45	7.97	1.56
Respiratory system (460–519)	30.6	0.79	0.00	2.88	0.26	18.2
Digestive system (520–579)	11.8	0.45	0.02	1.23	0.13	7.05
Genitourinary system (580–629)	1.51	0.01	0.00	0.11	0.00	0.89
Complications of pregnancy, childbirth and the puerperium (630–676)	0.04	0.06	0.07	0.05	0.00	0.05
Skin and subcutaneous tissue (680–709)	1.82	0.07	0.02	0.05	0.07	1.09
Musculoskeletal system and connective tissue (710–739)	1.53	4.07	26.9	8.80	20.8	3.66
Congenital anomalies (740–759)	0.43	1.50	1.04	1.29	0.78	0.85
Certain conditions originating in the perinatal period (760–779)	0.16	0.51	0.13	0.41	0.00	0.28
Symptoms, signs and ill-defined conditions (780–799)	14.0	0.90	0.47	2.99	0.52	8.59
Injury and poisoning (800–999)	0.90	4.47	47.0	5.25	41.9	4.19

*Abbreviations*: *CHM* Chinese herbal medicine, *COT* Chinese Orthopedic Traumatology, *ACU* Acupuncture

**Fig. 2:**
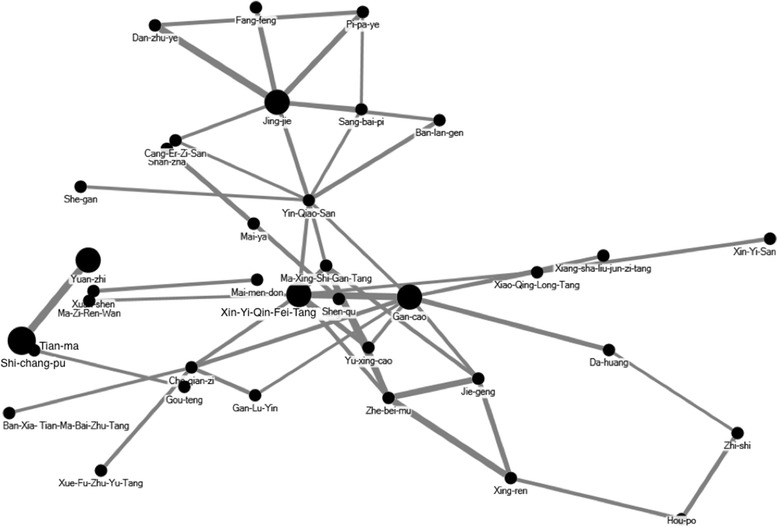
The core prescription pattern of Chinese herbal formulas and single herbs for children with cerebral palsy were analyzed through open-sourced freeware NodeXL. The thicker line width, defined as counts of connections between formulas and herbs, indicated the more significant prescription patterns in the network

To identify the prescription patterns of TCM doctors in treating children with CP, we also analyzed the Chinese herbal formulas and single herbs prescribed by TCM doctors (Table 4). The most commonly prescribed herbal formula was Ma-Zi-Ren-Wan (4.07%), followed by Liu-Wei-Di-Huang-Wan (3.34%) and Xiang-Sha-Liu-Jun-Zi-Tang (3.27%). The ten most common single herb prescribed by TCM doctors was Rhizoma Acori Graminei (Shi-chang-pu) (2.55%), followed by Radix et Rhizoma Rhei (Da-huang) (2.39%) and Rhizoma Gastrodiae (Tian-ma) (2.1%). The core patterns of Chinese herb medicine for the treatment of patients with CP included Xin-Yi-Qing-Fei-Tang and Radix Glycyrrhizae (Gan-cao) as well as Rhizoma Acori Graminei (Shi-chang-pu), Rhizoma Gastrodiae (Tian-ma) and Radix Polygalae (Yuan-zhi) (Fig. 2).

**Table 4:** The common TCM prescription for the treatment of children with cerebral palsy

Herbal formulas						
Pin-yin name	English name	Pin-yin name (Chinese materia medica name; botanical name)	N	%	Daily dose (g)	Average duration (day)
Ma-Zi-Ren-Wan	Hemp Seed Pill	Huo-ma-ren (Semen Cannabis; *Cannabis sativa* L.), Xing-ren (Semen Armeniacae; *Prunus armeniaca* L.), Bai-shao (Radix Paeoniae Alba; *Paeonia lactiflora* Pall), Zhi-shi (Fructus Aurantii Immaturus; *Citrus × aurantium* L.), Hou-po (Cortex Magnoliae; *Magnolia hypoleuca* Siebold & Zucc.), Da-huang (Radix et Rhizoma Rhei; *Rheum palmatum* L.), Feng-mi (honey)	2078	4.07	8.32	2.77
Liu-Wei-Di-Huang-Wan	Six Ingredient Pill with Rehmannia	Shu-di-huan (Radix Rehmanniae Preparata; *Rehmannia glutinosa (Gaertn.)* Libosch. ex Fisch. & C.A. Mey.), Shan-zhu-yu (Fructus Corni; *Cornus officinalis* Siebold & Zucc.), Shan-yao (Rhizoma Dioscoreae; *Dioscorea opposita* Thunb.), Fu-ling (Poria; *Wolfiporia cocos* (Schw.) Ryv. & Cilbn.), Mu-dan-pi (Cotex Moutan; *Paeonia suffruticosa* Andr.), Ze-xie (Rhizoma Alismatis; *Alisma orientale* (Sam.) Juz.)	1706	3.34	6.67	7.89
Xiang-Sha-Liu-Jun-Zi-Tang	Six Gentlemen Decoction with Aucklandia and Amomum	Mu-xiang (Radix Aucklandiae; *Aucklandia lappa* Decne.), Sha-ren (Fructus Amomi; *Amomum villosum* Lour.), Chen-pi (Pericarpium Citri Reticulatae; *Citri Reticulatae Pericarpium*), Ban-xia (Rhizoma Pinelliae; *Pinellia ternata* (Thunb.) Makino), Dang-shan (Radix Codonopsis; *Codonopsis pilosula* (Franch.) Nannf.), Bai-zhu (Rhizoma Atractylodis Macrocephalae; *Atractylodes macrocephala* Koidz.), Fu-ling (Poria; *Wolfiporia cocos*(Schw.) Ryv. & Cilbn.), Gan-cao (Radix Glycyrrhizae; *Glycyrrhiza uralensis* Fisch.), Sheng-jiang (Rhizoma Zingiberis Recens; *Zingiber officinale* Roscoe), Da-zao (Fructus Jujubae; *Ziziphus jujuba* Mill.)	1672	3.27	8.16	4.63
Shen-Ling-Bai-Zhu-San	Ginseng, Poria and Atractylodis Macrocephalae Powder	Bian-dou (Semen Lablab Album; *Lablab purpureus* (L.) Sweet), Ren-shen (Radix Ginseng; *Panax ginseng* C.A.Mey.), Bai-zhu (Rhizoma Atractylodis Macrocephalae; *Atractylodes macrocephala* Koidz.), Fu-ling (Poria; *Wolfiporia cocos*(Schw.) Ryv. & Cilbn.), Gan-cao (Radix Glycyrrhizae; *Glycyrrhiza uralensis* Fisch.), Shan-yao (Radix Paeoniae Alba; *Paeonia lactiflora* Pall), Lian-zi (Semen Nelumbinis; *Nelumbo nucifera* Gaertn.), Yi-yi-ren (Semen Coicis; *Coix lacryma-jobi* L.), Jie-geng (Radix Platycodonis; *Platycodon grandiflorus* (Jacq.) A.DC.), Sha-ren (Fructus Amomi; *Amomum villosum* Lour.), Da-zao (Fructus Jujubae; *Ziziphus jujuba* Mill.)	1588	3.11	7.58	4.4
Chai-Hu-Jia-Long-Gu-Mu-Li-Tang	Bupleurum plus Dragon Bone and Oyster Shell Decoction	Chai-hu (Radix Bupleuri; *Bupleurum chinense* DC.), Huang-qin (Radix Scutellariae; *Scutellaria baicalensis* Georgi), Ren-shen (Radix Ginseng; *Panax ginseng* C.A.Mey.), Ban-xia (Rhizoma Pinelliae; *Pinellia ternata* (Thunb.) Makino), Sheng-jiang (Rhizoma Zingiberis Recens; *Zingiber officinale* Roscoe), Da-zao (Fructus Jujubae; *Ziziphus jujuba* Mill.), Fu-ling (Poria; *Wolfiporia cocos* (Schw.) Ryv. & Cilbn.), Da-huang (Radix et Rhizoma Rhei; *Rheum palmatum* L.), Mu-li (Concha Ostreae), Qian-dan (Minium)	1219	2.39	9.58	3.7
Xin-Yi-Qing-Fei-Tang	Magnolia Flower Drink to Clear the Lungs	Xin-yi (Flos Magnoliae; *Magnolia biondii* Pamp.), Pi-pa-ye (Fol. Eriobotryae; *Eriobotrya japonica* (Thunb.) Lindl.), Zhi-zi (Fructus Gardeniae; *Gardenia jasminoides* J.Ellis), Zhi-mu (Rhizoma Anemarrhenae; *Anemarrhena asphodeloides* Bunge), Bai-he (Bulbus Lilii; *Lilium brownii* F.E.Br. ex Miellez), Huang-qin (Radix Scutellariae; *Scutellaria baicalensis* Georgi), Sheng-ma (Rhizoma Cimicifugae; *Cimicifuga foetida* L.), Mai-men-dong (Radix Ophiopogonis; *Ophiopogon japonicus* (Thunb.) Ker Gawl.), Shi-gao (Gypsum Fibrosum), Gan-cao (Radix Glycyrrhizae; *Glycyrrhiza uralensis* Fisch.)	1209	2.37	9.02	3.04
Shao-Yao-Gan-Cao-Tang	Peony and Licorice Decoction	Bai-shao (Radix Paeoniae Alba; *Paeonia lactiflora* Pall), Gan-cao (Radix Glycyrrhizae; *Glycyrrhiza uralensis* Fisch.)	1207	2.36	7.97	6.34
Ma-Xing-Shi-Gan-Tang	Ephedra, Apricot Kernel, Gypsum and Licorice Decoction	Ma-huang (Herba Ephedrae; *Ephedra sinica* Stapf), Xing-ren (Semen Armeniacae; *Prunus armeniaca* L.), Shi-gao (Gypsum Fibrosum), Gan-cao (Radix Glycyrrhizae; *Glycyrrhiza uralensis* Fisch.)	973	1.9	7.75	8.89
Xiao-Xu-Ming-Tang	Minor Extend Life Decoction	Ma-huang (Herba Ephedrae; *Ephedra sinica* Stapf), Chuan-xiong (Rhizoma Chuanxiong; *Ligusticum chuanxiong* S.H.Qiu, Y.Q.Zeng, K.Y.Pan, Y.C.Tang & J.M.Xu), Han-fang-ji (Radix Stephaniae Tetrandrae; *Stephania tetrandra* S. Moore), Xing-ren (Semen Armeniacae; *Prunus armeniaca* L.), Fang-feng (Radix Saposhnikoviae; *Saposhnikovia divaricata* (Turez.) Schischk.), Sheng-jiang (Rhizoma Zingiberis Recens; *Zingiber officinale* Roscoe), Ren-shen (Radix Ginseng; *Panax ginseng* C.A.Mey.), Zhi-fu-zi (Radix Aconiti Lateralis Preparata; *Aconitum carmichaeli* var. carmichaeli), Gui-zhi (Ramulus Cinnamomi; *Cinnamomum cassia* (L.) J.Presl), Bai-shao (Radix Paeoniae Alba; *Paeonia lactiflora* Pall), Huang-qin (Radix Scutellariae; *Scutellaria baicalensis* Georgi), Gan-cao (Radix Glycyrrhizae; *Glycyrrhiza uralensis* Fisch.)	968	1.89	4.09	9.73
Xiao-Jian-Zhong-Tang	Minor Construct the Middle (Burner) Decoction	Yi-tang (Maltose), Gui-zhi (Ramulus Cinnamomi; *Cinnamomum cassia* (L.) J.Presl), Bai-shao (Radix Paeoniae Alba; *Paeonia lactiflora* Pall), Sheng-jiang (Rhizoma Zingiberis Recens; *Zingiber officinale* Roscoe), Gan-cao (Radix Glycyrrhizae; *Glycyrrhiza uralensis* Fisch.), Da-zao (Fructus Jujubae; *Ziziphus jujuba* Mill.)	966	1.89	9.16	11.2
Single Herbs						
Pin-yin name	Chinese materia medica name	Botanical name				
Shi-chang-pu	Rhizoma Acori Graminei	*Acorus tatarinowii* Schott	2212	2.55	7.12	3.59
Da-huang	Radix et Rhizoma Rhei	*Rheum palmatum* L.	2067	2.39	7.71	1.99
Tian-ma	Rhizoma Gastrodiae	*Gastrodia elata* Blume	1822	2.10	7.19	1.50
Yuan-zhi	Radix Polygalae	*Dimocarpus longan* Lour	1778	2.05	7.89	1.17
Gan-cao	Radix Glycyrrhizae	*Glycyrrhiza uralensis* Fisch	1724	1.99	9.16	1.45
Zhe-bei-mu	Bulbus Fritillariae Thunbergii	*Fritillaria thunbergii* Miq.	1626	1.88	7.57	6.19
Xing-ren	Semen Armeniacae	*Prunus armeniaca* L.	1329	1.53	7.33	1.98
Hou-po	Cortex Magnoliae Officinalis	*Magnolia hypoleuca* Siebold & Zucc	1238	1.43	7.98	1.96
Jie-geng	Radix Platycodonis	*Platycodon grandiflorus* (Jacq.) A.DC	1223	1.41	6.72	1.52
Huang-qi	Radix Astragali	Astragalus membranaceus (Fisch.) Bunge	1059	1.22	9.14	1.71

In a previous study, we found that pediatric TCM users often visited TCM clinics due to the four common diseases: allergic rhinitis, dyspepsia, menstrual disorders, and musculoskeletal system and connective tissue diseases [18]. We further analyzed the prevalence of these four common diseases between children with CP who were TCM users and non-TCM users (Table 5). In all four diseases, the proportions of TCM users were significant higher (*p* < 0.0001) than non-TCM users. Regarding the medical expenditure, complementary TCM users had higher medical expenditure for utilizing outpatient clinical care. However, their medical costs for visiting ER and hospitalization were significantly lower than that of non-TCM user within one year of the diagnosis of CP (Table 6).

**Table 5:** Incidence rate ratio of diseases between non-TCM and TCM users

Disease	ICD-9-CM code	TCM user		Non-TCM user		Compared to non-TCM user
		N	%	N	%	IRR (95% CI)
Allergic rhinitis	477.9	3688	52.7	1329	31.5	1.67 (1.57–1.78)***
Dyspepsia and other specified disorders of function of stomach	536.8	2452	35.0	864	20.5	1.71 (1.58–1.85)***
Disorders of menstruation and other abnormal bleeding from female genital tract	626	600	8.58	215	5.09	1.68 (1.44–1.97)***
Disease of the musculoskeletal system and connective tissue	710–739	5034	72.0	1977	46.8	1.54 (1.46–1.62)***

*IRR* incidence rate ratio in Poisson regression

*** *p* value < 0.0001

**Table 6:** Total medical expenditure for utilizing ER service, outpatient clinical consultations and hospitalization within one year of diagnosis of cerebral palsy

Healthcare service	TCM user		Non-TCM user		
	Mean^a^	SD	Mean^a^	SD	*t*-test
ER	1671.7	1591.2	1976.1	1990.9	0.002
Outpatient clinical consultations	1739.4	2267.4	891.8	1327.0	0.03
Hospitalization	33342.7	63914.2	46583.5	80183.7	0.001

^a^New Taiwan dollars

## Discussion

This population-based study characterized complementary TCM usage among children with cerebral palsy in Taiwan. In this study, we found that the proportion of TCM users in children with CP (62.37%) was much higher than average (22.5%) [18]. Our study was in accordance with a previous study in which younger children with CP used complementary and alternative more frequently [24]. The lower the age of the child with CP, the higher the proportion of TCM utilization that was found. However, this finding was not in agreement with previous studies in which older children with allergic disorders were more likely to consult with TCM services [8–10]. Furthermore, while both TCM users and non-TCM users exhibited high rates of annual outpatient visits, TCM users showed a higher proportion than non-TCM users. Patients with brain damage need early and comprehensive medical management. In line with some studies’ reports on the benefits of treating brain damage or neurological disorders with TCM methods [15, 25, 26], it was quite common in Taiwan to use Chinese medicine to treat children with CP. Compared to other diseases, the utilization of TCM in patients with CP was much higher [18, 27, 28]. Possible reasons for this difference included the following: (1) parents desired a variety of ways to help their ill children, (2) parents’ thought that natural products such as Chinese herbs generally had fewer side effects for developing children, and (3) the NHI program covered high-quality Chinese medical outpatient care for children with CP [29, 30], ensuring low medical costs for TCM on the part of the patients.

Children with CP often suffer from other disorders of cerebral function, such as intellectual disabilities, neurodevelopmental disorders, epilepsy, visual disorders, and speech and hearing impairments [5]. However, chronic pulmonary disease is a leading cause of death among children with severe CP [31]. The causes of pulmonary disease in these children are recurrent aspiration caused by gastroesophageal reflux and palatopharyngeal incoordination and restrictive disease due to scoliosis [31]. This may have explained why the three most common causes of clinical visits for children with CP in both TCM and non-TCM groups were related to the nervous, respiratory and digestive systems.

Therefore, children with CP did not only suffer from neurological and musculoskeletal disorders, but the therapy for CP was based on patients’ limitations in body structure and function. While medication and/or surgery may have helped to reduce spasticity, hyperreflexia, and clonus, they did not improve weakness and incoordination [32, 33]. Moreover, it was uncertain whether these interventions enhanced functional outcomes. Some reports showed that injections of botulinum toxin type A (BTX A) in combination with physical and occupational therapy improved some functional outcomes [34, 35], but this treatment did not affect associated disorders such as gastrointestinal and pulmonary dysfunction. Therefore, patients and parents needed to seek other ways to solve these problems. Previous studies also found that children with multiple disabilities chose a wide range of treatments to complement conventional therapies [24, 36]. This may have been another reason for high TCM usage in children with CP.

In Taiwan, the acceptance of acupuncture among children is lower than Chinese herbal medicine [18]. Among the children with CP, we found that many of the patients received acupuncture or Chinese orthopedic traumatology for symptoms related to injury, musculoskeletal system and connective tissue disorder, mental disorder, and nervous system. The NHI program also fully covered the integrative approaches, including tuina massage (a kind of Chinese orthopedic traumatology methods), ear acupuncture, scalp acupuncture, and somatic acupuncture together to aim to improve the quality of life in children with CP [29]. This provided an option for these children to receive the acupuncture and Chinese orthopedic traumatology.

Among the most common Chinese herbal medicine prescribed for children with CP, majority of them were indicated to treat condition related to the digestive system. Children with CP often have growth failure, which is mainly associated with poor nutrition [37] due to inadequate intake and gastrointestinal abnormalities [37–41]. More than 90 percent of children with CP have clinically significant gastrointestinal symptoms such as swallowing disorders, chronic constipation, regurgitation and/or vomiting, chronic aspiration, and abdominal pain [41]. Furthermore, poor digestive function could lead to chronic pulmonary disease, the main cause of death in these patients [31]. The most commonly prescribed herbal formula in TCM to relieve constipation due to deficient fluid in the colon was Ma-zi-ren-wan, also known as Hemp Seed Pill. In a previous randomized double-blind study, Ma-zi-ren-wan was shown to be safe and effective at alleviating functional constipation [42]. One of the commonly prescribed single herb, Radix et Rhizoma Rhei, was also a commonly used laxative [43]. Other herbal formulas such as Liu-wei-di-huang-wan was used for improving osteoporosis [44] and Shao-yao-gan-cao-tang for relieving muscle spasm [45]. Since neurodevelopmental disorders [46], growth failure [37], orthopedic disorders [47, 48] and osteopenia [49–52] were commonly associated disorders in children with CP, it was reasonable that some of the TCM prescriptions were used for treating these illness. Another category of TCM prescriptions, such as Chai-hu-jia-long-gu-mu-li-tang [21], Rhizoma Acori Graminei [53], Rhizoma Gastrodiae [54] and Radix Polygalae [55], were used for alleviating spasms and regulate the central nervous system. These three herbs were included in the core patterns of Chinese herb medicine for the treatment of patients with CP. Children with CP were also commonly found to have spastic syndromes [56], dyskinetic syndromes [57], epilepsy [5] and emotional disorders [46]. Various Chinese herbs, such as Xin-Yi-Qing-Fei-Tang, Ma-Xing-Shi-Gan-Tang, Bulbus Fritillariae Thunbergii and Semen Armeniacae, were used for treating respiratory symptoms. Finally, Radix Astragali was used traditionally to raise Qi and has been found to modulate immunity [58]. Taken together, some of the commonly prescribed Chinese herbs were used to complement one another to improve the main symptoms of CP. Others could help treat associated disorders that were not improved by BTX A injection, physical or occupational therapy, such as gastrointestinal and pulmonary dysfunction.

With regard to the rate of TCM and non-TCM use among the four common diseases that we previously found to be prevalent in pediatric TCM users [18], musculoskeletal system and connective tissue diseases had the highest ratio in both groups. The main concerns of patients and parents were likely related to limitations of the body’s structure and function. In all four diseases, the proportion of TCM use was significantly higher (*p* < 0.0001) than non-TCM use. This was consistent with our previous study that showed that the rate of TCM use for these four common diseases was higher in children in Taiwan [18].

Interestingly, although the medical expenditure for visiting outpatient clinics within one year of diagnosis of CP of complementary TCM user were higher than the non-TCM users, the medical costs for utilizing ER service and hospitalization were significantly lower. Many parents and policy-makers concerned about the costs of complementary and alternative medicine for patients with CP [59, 60]. This study provided some substantial economic evaluation for the integration of TCM treatment into the clinical healthcare of CP.

Overall, our study provided useful information regarding healthcare and epidemiological patterns of TCM use to treat children with CP. The importance of this study was based on the following aspects: First, based on the literature review and our knowledge, this study was the first large-scale investigation of complementary TCM to patients with CP. Second, this study included all patients below age 18 in the NHIRD with catastrophic illness certificates of CP. The potential for selection bias was eliminated. Third, the NHI system provides low-cost and convenient medical insurance to nearly all residents in Taiwan. Under the NHI program, both Western- and Chinese-based medical resources are very accessible. In 2012, there were approximately 59,017 Western medical doctors and 5,556 licensed TCM doctors serving 23 million people in Taiwan. Moreover, 93.7% of Taiwan’s medical institutions, including hospitals and clinics, take part in the NHI program [61].

The present study had several limitations. First, the NHI system did not reimburse purchases of healthy foods containing herbal ingredients. Second, we were unable to estimate treatment efficacy and disease severity in this study due to the lack of disease severity data in the NHIRD. Lastly, the neuroscience evidence of acupuncture and pharmacological mechanism of Chinese herbal medicine remained unclear. Therefore, we expected future studies and clinical trials to investigate the mechanism and clinical efficacy based on this study.

## Conclusion

This study was a large-scale survey to characterize patterns of complementary TCM use among children with CP. The complementary use of TCM in children with CP was considerably high. The reasons for patients’ clinical visits were related to their neurodevelopmental and musculoskeletal disorders, as well as their respiratory and digestive system problems. Complementary TCM users had lower medical costs of utilizing ER service and hospitalization than non-TCM users. Future clinical trials and basic researches could be developed based on the findings of this study.

## Abbreviations

95% CIs: 95% confidence intervals
BTX A: Botulinum toxin type A
CP: Cerebral palsy
ER: Emergency room
NHI: National health insurance
NHIRD: National health insurance research database
RCIPD: Registry for catastrophic illnesses patient database
TCM: Traditional Chinese medicine.
